# Short Term Oral Methylergonovine Maleate Prophylaxis for Status Migrainosus. Case Series and Review of Literature

**DOI:** 10.3389/fneur.2019.00201

**Published:** 2019-03-20

**Authors:** Najiya Haque, Nauman Tariq

**Affiliations:** ^1^Department of Medicine, Fatima Memorial Hospital College of Medicine & Dentistry, Lahore, Pakistan; ^2^Headache Center, Johns Hopkins Medicine, Baltimore, MD, United States

**Keywords:** migraine, methylergonovine, methergine, status migrainosus, headache, dihydroergotamine

## Abstract

**Background:** Intravenous dihydroergotamine (DHE) is frequently used during inpatient hospitalizations or outpatient infusion therapies for 3–5 days in order to break the continuous cycle of status migrainosus. We tried a short term 7 days prophylaxis of oral methylergonovine after discharge in order to prevent status migrainosus relapse and extend the therapeutic benefit from IV DHE.

**Methods:** Patients were diagnosed with status migrainosus in clinic setting based on the ICHD-III criteria. They received 1 mg IV DHE every 8 h along with metoclopramide for 3–5 days followed by methylergonovine maleate oral tablets as prophylaxis for 7 days post discharge. They were asked to maintain their headache diaries which included data on headache frequency and intensity. A post discharge follow up at 1 and 68 weeks was planned. Clinical improvement was defined as >50% decrease in frequency and intensity of headaches. Intensity was graded on verbal numerical rating scale (VNRS) with 10 being the worst possible pain. The institutes IRB and ethics committee exempted this study from review given that it had only 3 patients.

**Results:** A total of 3 patients 25–45 years of age who benefited from IV DHE, consented to trial of Methylergonovine Maleate 0.4 mg oral tablets three times a day prophylaxis on the day of discharge for a period of 7 days. At 1 week post discharge, all of the 3 patients had reported sustained improvement with severity dropping from an average of 8/10 intensity to 3/10 on VNRS. The headaches frequency had dropped from daily to episodic in 2 of the 3 patients. At an average of 7 weeks post discharge, 2 out of the 3 patients had reported sustained benefit. The third patient relapsed to the pre-admission status migrainosus severity. One patient reported mild diarrhea and nausea but was still able to continue the drug for a week.

**Conclusion:** Methylergonovine maleate after 3–5 days of IV DHE infusions may be a feasible treatment strategy for status migrainosus. This approach has the potential to prolonged the benefit of IV DHE and prevent relapse in to status migrainosus.

## Introduction

Migraine has being ranked as third most prevalent disorder in the Global Burden of Disease Study 2010 accounting for at least 1.2 million visits to the emergency department in the US annually (1). Intractable status migrainosus can be difficult to treat in an outpatient setting and the relapse rate is high. Migraine patients frequently revisit the emergency room at least once within a 6 month period, 9% of revisits within 72 h, and 46.2% within 90 days of the index migraine visit (1). ICHD-III criteria defines status migrainosus as a debilitating migraine with or without aura lasting over 72 h that cannot be better accounted for by another ICHD-3 diagnosis. A multitude of drugs are available for the treatment of migraines, intravenous DHE is frequently used for status migrainosus (2–4). The use of methysergide and its active metabolite methylergonovine has been extensively studied (5, 6). We believe that methylergonivine has the potential to prolong the benefit of IV DHE as well as prevent relapse into status migrainosus. Therefore, we prospectively collected a case series of 3 patients in order to assess safety and short term post discharge outcomes.

### Case 1

The patient having developed migraine headaches since teenage years presented to the headache clinic with complaints of persistent bilateral throbbing headaches with photophobia, phonophobia, and nausea on a constant daily basis for last 4 months fulfilling the ICHD-III criteria for status migrainosus. As an outpatient tried short term intranasal DHE, triptans, and NSAIDs to break her cycle of headaches but was unsuccessful. The decision was made to admit the patient to the inpatient unit where IV DHE infusions every 8 h were started. By the end of the second day of infusion, at the time of the 6th dose of IV DHE, headache severity on VNRS dropped from 8/10 to 3/10. On the 4th day, the patient was discharged with a headache severity level of 1/10. Patient was started on methylergonovine maleate tablets 0.4 mg dose TID for 7 days. One week post discharge maintained a headache severity level of 1/10 with 2 headache free days out of 7. At 6 weeks follow up clinic visit there was continued >50% improvement in terms of headache intensity. The patient reported at least 10 headache free days in the last 7 weeks.

### Case 2

A patient with major depression who has had a long standing history of migraine with and without aura since late teens; headaches gradually transformed in to status migrainosus over a span of few months for unclear reasons. MRI brain did not show any acute pathology. The patient had tried and failed outpatient trials of oral prednisone burst therapy for 7 days, oral naratriptan for 4 days. Nortriptyline, topiramate, and nadolol were also tried for several months without any benefit. Patient had also failed on botulinum toxin treatment twice. At the time of hospital admission for IV DHE the patient had been in status migrainosus for at least 2 years. Headache severity on VNRS at the time of inpatient admission was 8/10. IV DHE was started 8 h. Headaches were resistant to the initial doses of IV DHE, however by the morning of the 4th day, headache severity had slightly dropped to 6/10. The patient was discharged on methylergonovine tablets for 7 days. One week post discharge clinic visit assessment revealed occurrence of moderate severity headaches at an average of 6/10, which was mild interval improvement as compared to pre-admission pain state. Nausea and diarrhea attributed to methylergonovine were reported however medication was not stopped. At 7 weeks post discharge, patient reported that pre admission severity had returned with no headache free days in the interim.

### Case 3

Patient presented to the headache clinic with a diagnosis of episodic migraine without aura which started 7 years ago. Episodic headache attacks lasting for 1–3 days occurring on average 5–6 times a month. Both NSAIDS and triptans were helpful in aborting these headaches. Patient started a new job that aggravated underlying anxiety and stress. Her headaches became more frequent and evolved into a chronic migraine lasting for at least 15 days a month. Topiramate, beta-blocker, and antidepressant class of medicines had been tried as a prophylactic on separate occasions, but the patient either developed side effects or the drugs were deemed ineffective. At the time of inpatient hospital admission patient was having continuous daily migraine headaches for at least 3 months, missing work on average 1 day per week. At the time of admission headache severity on VNRS was 8/10. IV DHE protocol with every 8 h infusions was started. By the 3rd day, headaches had been completely aborted. This was followed by methylergonovine prophylaxis for 7 days. One week post discharge visit, only one migraine attack was reported which was aborted by a combination of NSAID and triptan within 4 h of onset. At 8 weeks follow up clinic visit, there had been sustained improvement in headache frequency and severity. An average of 1 migraine attack per week aborted by her NSAIDS and triptans was reported.

## Discussion

We have described three cases of status migrainosus patients with age groups ranging from 25 to 45 years, two of whom responded significantly well to IV DHE followed by a 7 days oral prophylaxis of methylergonovine maleate. The third patient also partially responded to IV DHE and oral methylergonovine combination but unfortunately relapsed back to status migrainosus 7 weeks after discharge. Only one patient developed mild diarrhea and nausea but not severe enough to discontinue the drug.

Chronic daily headaches majority of which are migraine can be present in up to 4% of the general population (7). Inpatient admission is often needed to treat patients with intractable migraine who have failed outpatient management. Nagy et al. studied the use of IV DHE for inpatient management of refractory primary headaches; concluding that IV DHE for up to 5 days may have a cumulative effect for up to a month as compared to shorter courses (8). Patients who have chronic migraine associated with medication overuse after successful treatment have shown a relapse rate of as high as 40% (9). The preventive management of migraine can be seen as originating from the development of methysergide in the 1960s (10). Methylergonovine is the active metabolite of Methysergide and is 40 times more potent (11). It is a semisynthetic ergot alkaloid widely used in the treatment of postpartum hemorrhage (12, 13) as well as the prophylaxis of migraines (13). It acts as an agonist for 5HT1 receptors (6, 13) and antagonist of 5HT2 receptors on the temporal artery (11). Saxena et al. demonstrated the drug's selective vasoconstriction of arteriovenous anastomosis in the carotids via 5-HT2 receptor activation (14). It also binds to noradrenaline α2A, α2B, and α1 receptors, and dopamine D2L and D3 receptors. There have been studies proving the efficacy of methysergide in the treatment of migraines as well as cluster headaches (4, 15). The first clinical trial was performed by Sicuteri 2 using 18 cases, 10 resistant to all other treatment and 2 with cluster headache. Fifteen patients were given oral 8 mg methysergide daily for 5 days, followed by 4 to 6 mg daily for 15–60 days. Nine patients became headache free and the attacks decreased in frequency or duration in the others. In review, Graham studied 500 headache patients over the course of 4,276 patient months; reporting ~70 per cent showed decreased frequency and severity of migraines and cluster headache (16).

Long term use of this drug has also been implicated in the development of pleural, cardiac valvular, aortic, and retroperitoneal fibrosis (17). As a serotonin antagonist, methysergide increase endogenous serotonin which could cause fibrosis analogous to carcinoid syndrome (18). The estimated incidence of fibrosis in patients taking methylergonovine maleate is 1 in every 5,000 (18, 19). Graham studied 1,000 patients from a 5 year period, 1% of whom developed retroperitoneal fibrosis. Since 1965, 27 patients have been reported to have developed retroperitoneal fibrosis after being treated with daily 2 mg to 28 mg dose for a time span of 9 and 54 months (18). Partial or complete receding of fibrosis has also been reported within 3 months of stopping the drug in some cases (17, 18).

## Conclusions

Based on the above results, we believe that a short term oral prophylaxis with Methylergonovine maleate after 3–5 days of IV DHE infusions may be a safe and feasible treatment strategy. It has the potential to prevent readmission secondary to status migrainosus relapse. Although the data was collected prospectively, the low power of this study carries a risk of skewed results. Another limitation was a relatively short follow up time period and whether the benefit resulted from the IV DHE alone. A prospective controlled study with a control arm could further validate these results.

## Author Contributions

NT director of the headache center, collected data. NH wrote and compiled the manuscript.

### Conflict of Interest Statement

The authors declare that the research was conducted in the absence of any commercial or financial relationships that could be construed as a potential conflict of interest.
